# Caloric restriction in female reproduction: is it beneficial or detrimental?

**DOI:** 10.1186/s12958-020-00681-1

**Published:** 2021-01-04

**Authors:** Jiayi Sun, Xin Shen, Hui Liu, Siying Lu, Jing Peng, Haibin Kuang

**Affiliations:** 1 Department of Physiology, Basic Medical College, Nanchang University, Nanchang, Jiangxi 330006 People’s Republic of China; 2 Department of Clinical medicine, School of Queen Mary, Nanchang University, Nanchang, China; 3 Department of Gynecology, Nanchang HongDu Hospital of Traditional Chinese Medicine, 264 MinDe Road, Nanchang, Jiangxi 330006 People’s Republic of China; 4 Jiangxi Provincial Key Laboratory of Reproductive Physiology and Pathology, Medical Experimental Teaching Center of Nanchang University, Nanchang, China

**Keywords:** Caloric restriction, Undernutrition, Reproduction, Energy, Female

## Abstract

Caloric restriction (CR), an energy-restricted intervention with undernutrition instead of malnutrition, is widely known to prolong lifespan and protect against the age-related deteriorations. Recently it is found that CR significantly affects female reproduction via hypothalamic (corticotropin releasing hormone, neuropeptide Y, agouti-related peptide) and peripheral (leptin, ghrelin, insulin, insulin-like growth factor) mediators, which can regulate the energy homeostasis. Although CR reduces the fertility in female mammals, it exerts positive effects like preserving reproductive capacity. In this review, we aim to discuss the comprehensive effects of CR on the central hypothalamus-pituitary-gonad axis and peripheral ovary and uterus. In addition, we emphasize the influence of CR during pregnancy and highlight the relationship between CR and reproductive-associated diseases. Fully understanding and analyzing the effects of CR on the female reproduction could provide better strategies for the management and prevention of female reproductive dysfunctions.

## Introduction

Caloric restriction (CR) is a dietary intervention that restricts the energy intake and induces undernutrition without malnutrition [1]. CR is also termed energy restriction/deficiency, food restriction, dietary restriction and negative energy balance [1, 2]. In the 1930s, McCay et al. [3] first discovered that CR increased the lifespan of rats who were restricted in food intake at the weaning or 2 weeks after the weaning. To date, CR is generally considered to prolong the mean as well as maximum lifespan and delay age-related deleterious alternations in diverse species, from yeast to mammals [1, 4].

Recently a hypothesized explanation of CR longevity-extending effect, which is based on the disposable soma theory of aging, is that energy resource is reallocated from reproduction to somatic maintenance [5, 6]. Indeed, CR inhibits reproductive functions for long life in both sexes of invertebrates and vertebrates, and this effect is significantly stronger in laboratory model species. It is demonstrated that the reproductive traits with more energy expenditure suffer higher reductions. In most experiments, females are exposed more reproductive costs than males under CR, so females suffer a larger and more significant elongation in lifespan than males [6].

It is well-known that CR impairs female reproduction, but CR can also benefit it. Selesniemi et al. [7] reported that adult-onset CR enables to maintain activities of reproductive axis in aged female mice. Nowadays, more and more obese even normal-weight women go on a diet to achieve a beautiful figure. Therefore, it is necessary to have a systematic understanding that whether CR induced by dieting is favorable or harmful on female reproduction. In this review, we discuss the effects of CR in hypothalamus-pituitary-ovarian (HPO) axis, ovary and uterus. In addition, we investigate the influence of CR during pregnancy and highlight the potential role of CR in female reproductive-associated diseases.

## The roles of CR in HPO axis

### Coordination of HPO axis with mediators controlling energy homeostasis

**Fig. 1:**
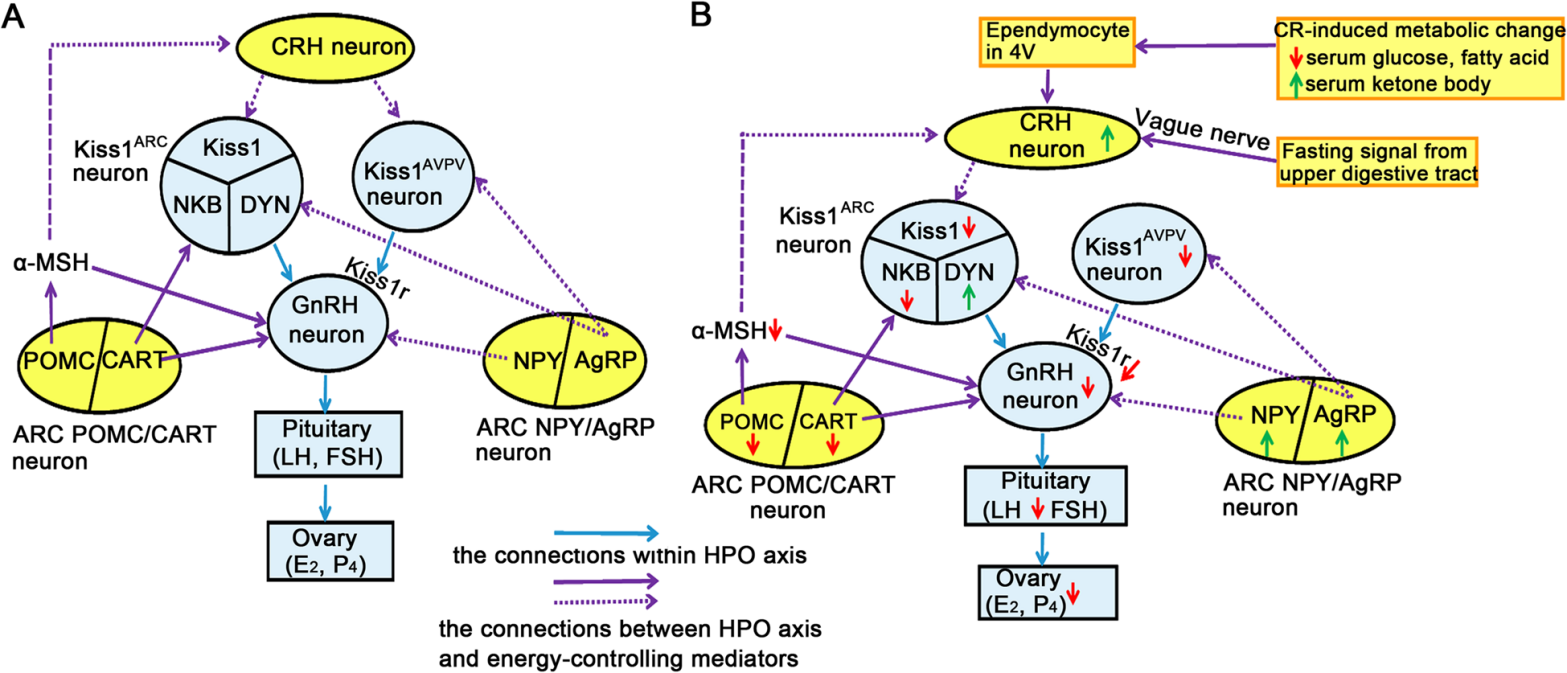
The possible interaction between HPO axis and hypothalamic neurons controlling energy homeostasis in rodents. Schematic representation of possible interaction between HPO axis (blue circles and rectangles) and hypothalamic neurons (yellow circles) controlling energy homeostasis in normal energy status and CR. **a** In normal energy status, the CRH neurons and orexigenic NPY/AgRP neurons inhibit HPO axis while anorexigenic POMC/CART neurons activate HPO axis. **b** CR finally suppresses HPO axis by activating NPY/AgRP and inhibiting POMC/CART neurons. During the CR, low serum glucose and fatty acid, high serum ketone body and fasting signals from upper digestive tract activate A2 noradrenergic neurons in NTS. Therefore, the adrenergic input from NTS stimulates CRH neurons and thus inhibits Kiss1^ARC^. Solid arrow indicates the promotion. Dotted arrow indicates the inhibition. The green arrow indicates upregulation while the red arrow indicates downregulation under CR

**Fig. 2:**
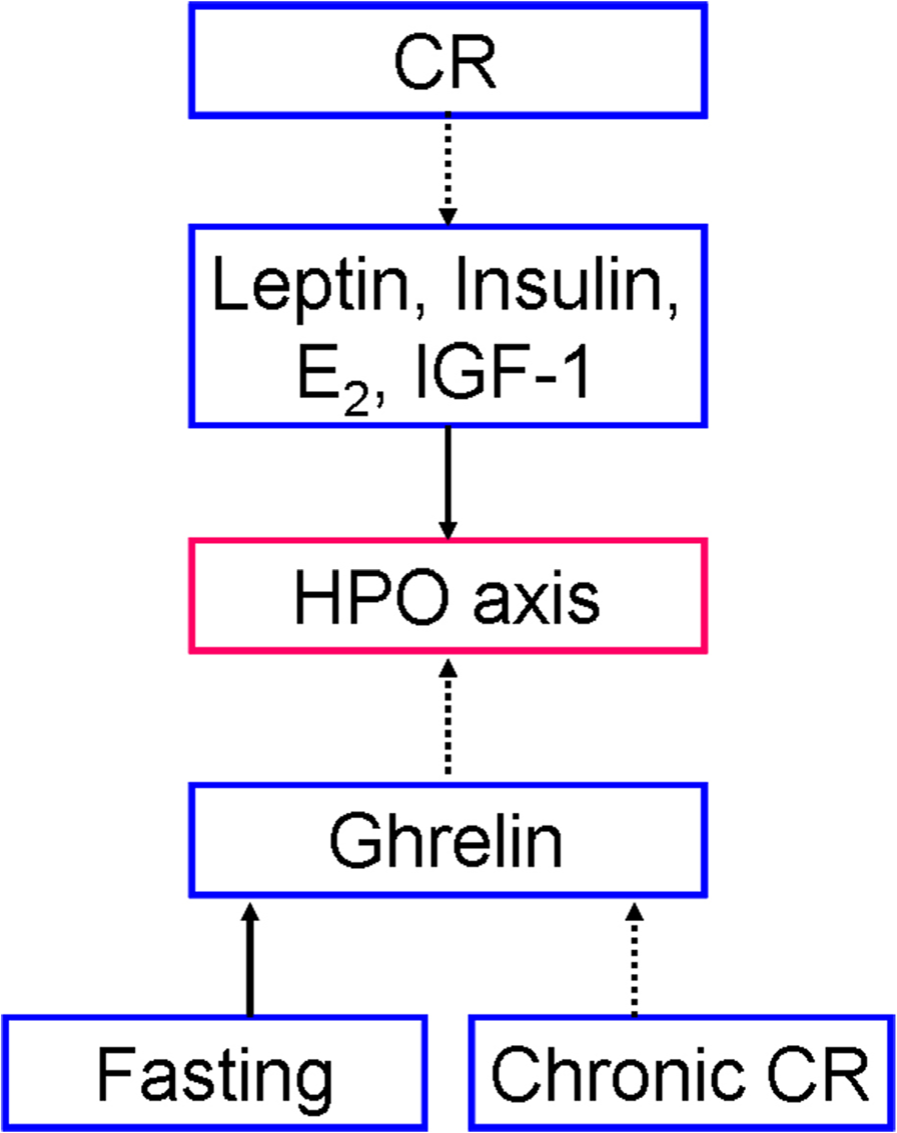
The interaction between peripheral hormones and HPO axis. The anorexigenic factors such as leptin, insulin, estradiol (E_2_) and insulin-like growth factor (IGF-1) activate HPO axis while the orexigenic ghrelin inhibits HPO axis. CR downregulates the expressions of leptin, insulin, E_2_ and IGF-1, which will lead to the inhibition of HPO axis. Ghrelin expression is upregulated during fasting but it is downregulated during chronic CR. Solid arrow indicates the promotion. Dotted arrow indicates the inhibition

In all examined mammals, two main hypothalamic populations of kisspeptin (*kiss1*) neurons localize in caudal arcuate nucleus (ARC) and rostral preoptic area (POA). ARC kisspeptin neuron (Kiss1^ARC^) is also referred as KNDy neuron because it co-expresses the positive autoregulator neurokinin B (NKB) and the negative autoregulator dynorphin (DYN). Kisspeptin neurons in both ARC and POA positively innervate GnRH neurons via kiss1 receptor (*kiss1r*). As following studies are predominantly based on laboratory rodents, we just discuss the differences of kisspeptin neurons between rodents and humans. One difference is that the rostral population in rodents is collectively located in the rostral periventricular area of the third ventricle (RP3V), which consists of the anteroventral periventricular nucleus (AVPV) and the periventricular nucleus (PeN). The POA kisspeptin neurons in humans reside more dispersedly. The other one is that in rodents, Kiss1^ARC^ is implicated in negative feedback of estrogen while AVPV kisspeptin neuron (Kiss1^AVPV^) is implicated in positive feedback. In contrast, both negative and positive feedback are mediated by Kiss1^ARC^ in humans [8, 9]. Both hypothalamic (CRH neurons, ARC NPY/AgRP neurons, ARC POMC/CART neurons) and peripheral (leptin, insulin, ghrelin, IGF-1) mediators are response to energy balance, and their relationships with HPO axis are shown in Figs. 1 and 2 respectively. Noticeably, kisspeptin neurons are the critical hubs of these linkages.

#### Hypothalamic mediators

Figure 1a indicates that some metabotropic neurons in hypothalamus enable to regulate HPO axis. Corticotropin releasing hormone (CRH) neurons in hypothalamus of adult female rats directly inhibit Kiss1^ARC^ and Kiss1^AVPV^ through CRH receptors [10]. The orexigenic neuropeptide Y (NPY)/agouti-related peptide (AgRP) neurons in ARC are negative to HPO axis. Padilla et al. [11] discovered that AgRP neurons in mice give inhibitory innervation to Kiss1^ARC^ and Kiss1^AVPV^, but they do not give any neurotransmitter or neuropeptide to GnRH neurons. Although GnRH neurons in female rodents express both stimulatory NPY Y4 receptors and inhibitory Y1 receptors, the latter is responsible for the major effect of NPY peptide on GnRH neurons [12, 13]. GnRH neurons in adult rats also express inhibitory NPY Y5 receptors [14]. The anorexigenic pro-opiomelanocortin (POMC)/cocaine- and amphetamine-regulated transcript (CART) neurons in ARC are positive to HPO axis. In female mice, the excitatory effect of POMC neurons to GnRH neurons is predominantly mediated by the POMC-cleaved product α-melanocyte stimulating hormone (α-MSH), which excites GnRH neurons via both melanocortin receptor 3 (MC3R) and MC4R [12]. However, α-MSH inhibits CRH neurons via MC4R [15]. The experiment in female mice found that AgRP peptide, which is an antagonist of melanocortin receptors, attenuates the MC4R-mediated activation on GnRH neurons [16]. Interestingly, POMC neurons in female mice negatively innervate NPY/AgRP neurons and this innervation is enhanced by estradiol (E_2_) [17]. It has been discovered that CART postsynaptically depolarize Kiss1^ARC^ and GnRH neurons in female rats [18]. Collectively, in normal energy status, CRH neurons and ARC NPY/AgRP neurons inhibit HPO axis while ARC POMC/CART neurons activate HPO axis.

#### Leptin

Leptin is an adipocyte-derived anorexigenic factor. The stimulatory effect of leptin on HPO axis is dominant at hypothalamic level. Leptin directly activates Kiss1^ARC^ in mice, sheep and guinea pigs. It is summarized that leptin deficiency in mice decreases not only ARC *kiss1* mRNA level but also the amounts of Kiss1^AVPV^ [19]. Although GnRH neurons do not express leptin receptors (LepRs) [19–21], leptin in rodents can indirectly stimulate them through the neurons in hypothalamic premammillary nucleus (PMV) [22]. Generally, ARC POMC/CART neurons express facilitatory LepR while ARC NPY/AgRP neurons express inhibitory LepR [15]. Indeed, leptin can exert female-specific stimulatory effect on GnRH neurons via CART in adult rats [14]. However, strong evidences from laboratory rodents indicate that NPY-Y1/Y5 receptor signaling [2, 14] and MC3R/MC4R-mediated signaling [21] are leptin-independent.

#### Ghrelin

Ghrelin is the only circulating stomach-derived peptide and it functionally antagonizes leptin [23]. Ghrelin predominantly inhibits HPO axis [23–25] through following 3 approaches: (i) Ghrelin directly inhibits Kiss1^AVPV^ [24] and GnRH neurons [25] in female rats. (ii) Ghrelin promotes the release of CRH in female rhesus monkeys, so it can indirectly repress GnRH neurons [23, 26]. (iii) Ghrelin stimulates NPY neurons and subsequently inhibits POMC neurons in rodents [27]. Although ghrelin primarily suppresses gonadotropin secretion in female animals and women [28], it benefits basal luteinizing hormone (LH) and follicle-stimulating hormone (FSH) secretion in female rats [25]. Ghrelin in mouse placenta negatively modulates early embryonic development [23].

#### Insulin

The anorexigenic insulin is found to activate HPO axis. Although in vitro mice study discovered that insulin can directly modulate GnRH neurons, in vivo studies of adult ewes and mice gave an opposite evidence [29, 30]. Indeed, insulin activates Kiss1^ARC^ in mice via functional insulin receptors [31]. Also, in laboratory animals, insulin excites ARC POMC neurons and suppresses NPY/AgRP neurons [30]. In addition, insulin in mice directly stimulates gonadotropes to enhance the LH mRNA expression [32].

#### IGF-1

The hepatic insulin-like growth factor-1 (IGF-1) enables to activate HPO axis. Firstly, both intracerebroventricular-infused and peripherally injected IGF-1 in the prepubertal female rodents can directly activates Kiss1^AVPV^ and GnRH neurons, thus leading to a precocious puberty [29]. Secondly, experiment in female rats demonstrated that low circulating IGF-1 caused by CR inhibits the pituitary gonadotropes, therefore represses the secretion of LH, FSH and thus estrogen [33]. Thirdly, IGF-1 signaling in ovine induces the activation of primordial follicles [34]. In addition, IGF-1 in mammalian ovary stimulates steroidogenesis, either alone or in synergy with gonadotropins [35].

### CR-induced alternations negatively affect HPO axis

It is found that negative energy balance in female mammals inhibit HPO axis by suppressing pulsatile GnRH secretion and then attenuating pulsatile LH release from pituitary, resulting in infertility [2, 20, 36]. Noticeably, the experiments in adult female rodents discovered that the extent of this inhibition is different between acute fasting and chronic CR. The former marginally inhibits HPO axis because it hardly changes KNDy-related gene expression, but it suppresses *Kiss1r* expression on GnRH neurons. The latter totally inhibit HPO axis because it not only decreases ARC *kiss1*, NKB, AVPV *kiss1* and *kiss1r* expression but also increases DYN expression [20, 36]. Under CR, the diverse changes in both central and peripheral regulators contribute to HPO axis inhibition.

Figure 1b demonstrates that alternations in hypothalamic mediators inhibit HPO axis under CR. Initially, a series of rodent experiments discovered that CR activates AgRP neurons [11] and increases NPY mRNA level [37]. Also, CR decreases the expression of POMC [2] and CART [18]. Therefore, the activation of ARC NPY/AgRP neurons and the suppression of ARC POMC/CART neurons enable to inhibit HPO axis. Secondly, studies in laboratory animals (mainly rodents) found that there are two avenues that finally activate CRH neurons. One is that ependymocytes in the fourth ventricle (4 V) sense CR-induced high ketone-body availability and low glucose as well as fatty acid availabilities. Then these ependymocytes send the energy-deficient information to A2 noradrenergic neurons in solitary tract nucleus (NTS) [38, 39]. The other pathway is that fasting signals from upper digestive tract stimulate NTS A2 noradrenergic neurons via vague nerve [39]. Converging these two pathways, CRH neurons that are received the stimulatory input from A2 noradrenergic neurons [38, 39] release high level of CRH and thus inhibits Kiss1^ARC^ [40]. Interestingly, Deura et al. [40] discovered that in female rats, the CR sensor ependymocytes may also stimulate A6 noradrenergic neurons in NTS and then activate CRH neurons. These CRH neurons inhibit Kiss1^AVPV^.

Fig. 2 demonstrates that alternations of peripheral hormones inhibits HPO axis under CR. In mammals (mainly rodents), CR decreases serum leptin [15], insulin [2, 41] and insulin-like growth factor-1 (IGF-1) [2, 33, 41]. This series of hormonal changes enable to repress pulsatile LH secretion, therefore contributing to the inhibitory effect of CR on HPO axis [2, 15, 20, 33]. However, a strong evidence from adult female rats shows that hypoleptinaemia is not the crucial signal leading to the inhibition of ARC *kiss1* and LH during CR [20]. Although serum ghrelin is decreased during fasting, it is increased in chronic CR [2]. In ovariectomized estrogen-replaced rats, fasting-induced hyperghrelinaemia suppresses pulsatile LH secretion [24]. In addition, CR decreases plasma E_2_ in rodents and ruminants [2, 42–45], which is consistent with HPO axis inhibition. Interestingly, CR enhances the negative feedback (mice) [2, 36] and attenuates the positive feedback (rhesus monkeys) [46] of E_2_ on HPO axis. It is also found that E_2_ as an anorexigenic factor enables to inhibit ARC NPY/AgRP neurons and activate ARC POMC neurons [31]. Therefore, low E_2_ level caused by CR also contributes to the inhibition of HPO axis. Intriguingly, chronic CR in adult female rodents reduces serum LH in the presence of estrogen [36, 42] but increases serum LH in the absence of estrogen [36, 47], suggesting that the existence of estrogen is necessary in the effect of CR on HPO axis.

### CR delays the onset of female puberty

Puberty is started by re-awaking the GnRH pulse generator that is dormant before [29, 48]. Recent experiments have discovered that pubertal timing in female mammals is delayed by CR [30, 48], and it is restored once ad libitum (AL)-feeding was resumed [21]. It is generally accepted that hypothalamic *kiss1* is a gatekeeper of puberty [19, 49]. Therefore, reduced hypothalamic *kiss1* expression during prepubertal period may be the key mechanism of CR deferring puberty onset.

The changes in brain under CR delay the timing of puberty. The experiment in immature female rodents found that hypothalamic AMP-activated protein kinase (AMPK)-kisspeptin signaling regulates puberty onset. Hypothalamic AMPK, which can sense whole-body energy status, is found to be activated (i.e. phosphorylated) by CR and thus postpone the onset of puberty. More specifically, CR increases pAMPK level in Kiss1^ARC^ and thus suppresses ARC *kiss1* gene expression. However, the effect of hypothalamic pAMPK on Kiss1^AVPV^ is not discovered [50]. In addition, the experiment in female rats discovered that CR defers pubertal maturation by attenuating NKB-neurokinin-3 receptor (NK3R) signaling [51] as NKB is a positive autoregulator of Kiss1^ARC^.

Studies in rodents and humans demonstrated that leptin is just a permissive factor of pubertal onset because it alone cannot advance the onset of puberty [19]. It has been discovered that the suppression of leptin/LepR-kisspeptin/*Kiss1r*-GnRH signaling in female rats mediates the inhibitory effect of CR on puberty onset [48]. The novel leptin-α-MSH-kisspeptin -GnRH pathway in rats and mice is a possible mechanism of pubertal delay caused by CR [52]. High serum ghrelin can also delay puberty onset, but female rats are less sensitive to the effect of ghrelin than males [23]. It is discovered that the production of hypothalamic pAMPK is repressed by anorexigenic signals (e.g. leptin, insulin and E_2_) while is induced by orexigenic signals (e.g. ghrelin) [53], so it may be a considerable method for CR to defer pubertal timing.

## The roles of CR in ovary

### The roles of CR in folliculogenesis

Originally, studies of female rodents with CR initiated at weaning have proved that CR extended reproductive lifespan. However, CR during ablactation also impeded adolescent growth and sexual maturation, which interfered experiments [54]. Fortunately, recent studies of female rodents with adult-onset CR effectively excluded these interference factors. These experiments discovered that CR delays ovary aging through the maintenance of ovarian oocyte-containing follicle reserve [7, 42, 55–59] and good egg quality [7, 60, 61]. Although CR reduces fertility, it retains reproductive capacity and prolongs the reproductive lifespan. Therefore, when CR rodents are returned to AL feeding, their reproductive performances (i.e. fertility, fecundity and postnatal offspring survival rate) rebound or are even higher than that in AL condition [7].

#### CR benefits follicle pool reservation

The maintenance of follicle pool can reduce fertility and prevent premature ovarian failure [7, 57]. Compared with AL control group, CR in adult female rodents significantly increased the number of primordial follicles (PMFs). This finding indicates that CR reduces the rate of PMF activation, thus inhibiting the transition from primordial follicle to primary follicle [7, 42, 55–59]. Secondly, the number of secondary follicles, antral follicles and corpus luteum were dramatically lower in CR-fed rodents. This observation suggests that CR suppresses the ovarian follicle development at different stages, follicle maturation and ovulation [39, 51, 53, 54]. CR inhibits follicle atresia because CR-fed mice and rats had the significantly low amount of atretic follicles [7, 42, 56, 57]. Also, CR inhibits the total follicle loss as the dramatically increased number of total surviving follicles was seen in CR-fed rodents [57–59]. Although low fertility is observed in CR-fed mice, the capacity of fertility is augmented. Therefore, the fertility rebounds once AL-feeding is resumed [7]. It is noticed that CR also can augment the follicle pool and elongate the ovarian lifespan in adult female rats treated with chemotherapy [4]. SIRT1720, which partially mimics CR, achieves the similar effect in high-fat diet-induced obesity [58].

**Fig. 3:**
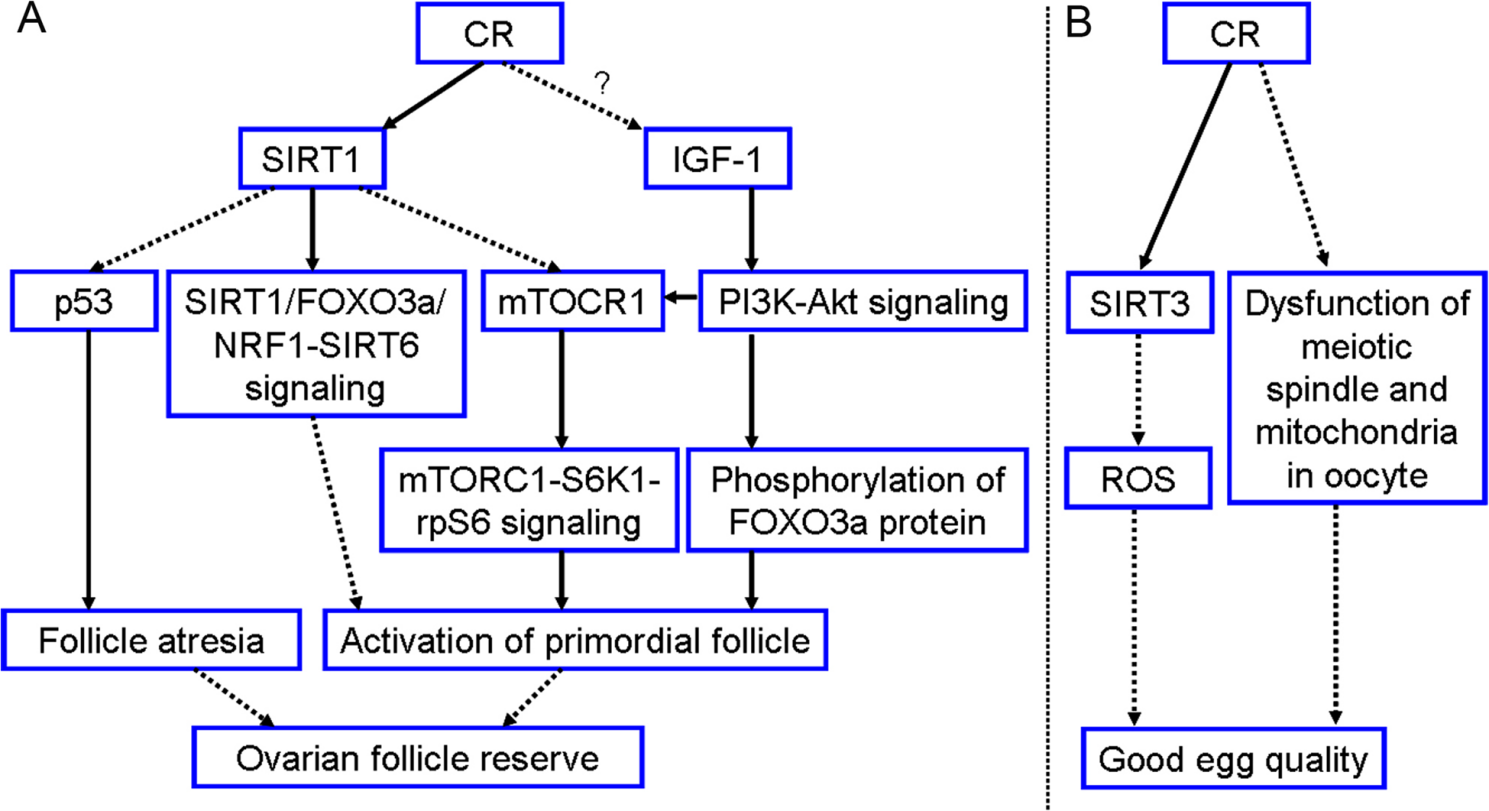
The potential mechanism of CR delaying ovary aging in female rodents. This mechanism is divided into two avenues. **a** One is CR preserving ovarian follicle pool, which is mediated by the SIRT1 activation and IGF-1 inhibition, which still need to be elucidated. **b** The other one is CR increasing egg quality, which is achieved by activating SIRT3 and inhibiting the occurrence of meiotic spindle and mitochondria disorder. Solid arrow indicates the promotion. Dotted arrow indicates the inhibition

Figure 3a shows the mechanism that CR increases ovarian follicle pool. Initially it is found that CR enhances SIRT1, FOXO3a, NRF1 and SIRT6 gene expression in rodent ovary. More specifically, SIRT1, FOXO3a and SIRT6 are predominantly expressed in the oocytes and hardly expressed in the granulosa cells. Due to SIRT1-FOXO3a-NRF1 complex formed on the SIRT6 promoter can upregulate SIRT6 expression, activation of SIRT1/FOXO3a/NRF1-SIRT6 signaling is one of the avenues which CR hinders the transition from PMFs to primary follicles [57–59, 62, 63]. SIRT1 upregulation by CR is important because it can also downregulate both p53 [53, 54] and mTOR complex 1 (mTORC1) [42, 58, 63] gene expression in rodent ovary. The evidence that low p53 inhibits follicle atresia is support by following studies: (i) p53 protein in rats is expressed in the apoptotic granulosa cells of atretic follicles [64]. (ii) Reduced p53 level in rat ovary is related to a significant decrease in the amount of apoptotic granulosa cells as well as atretic follicles [65]. (iii) p53 in mice is implicated in the regulation and selection of oocytes at checkpoints, such that oocytes that would otherwise be lost may persist when p53 is absent or reduced [66]. Recent studies from rodent models discovered that SIRT1 suppresses mTORC1-p70S6 kinase (S6K1)-ribosomal protein S6 (rpS6) signaling, thus preserving PMFs in quiescent state [42, 58, 63]. The most critical intra-oocyte signaling that controls PMF activation is the PI3K-Akt signaling. CR in female mice inhibits PI3K-Akt signaling and subsequently represses FOXO3a phosphorylation. The non-phosphorylated FOXO3a proteins are remained in oocyte nucleus, culminating in sustaining quiescent PMFs and thus maintaining ovarian follicle pool [55, 67]. Interestingly, it is found that CR preserving PMF pool is associated with low IGF-1 in rat ovary [33], and IGF-1 indeed activates PMFs via PI3K-Akt pathway in sheep ovary [34]. Therefore, CR may preserve PMF pool of rats by inhibiting IGF-1-PI3K-Akt signaling. It is also summarized that this signaling can upregulate mTORC1 expression [68]. In addition, CR overexpressing IGF-1 receptors (IGF-1Rs) may mediate the inhibition of follicle atresia because IGF-1Rs enable to antagonize cell apoptosis [33].

#### CR benefits egg quality

Two experiments in adult female mice give compelling evidence that CR enables to overcome the aging-related deterioration of egg quality: (i) The fecundity and postnatal offspring survival rate were remarkably increased in CR-then-AL fed mice [7]. (ii) The aging-related increases in aneuploidy, chromosomal misalignment on the metaphase plate, meiotic spindle abnormalities, mitochondrial aggregation and decreased ATP level, which were occurred in oocyte of AL-fed mice, were not exhibited in age-matched CR mice [61]. Therefore, good egg quality maintained by CR has a beneficial effect on oocyte meiotic maturation and fertilization, pre-implantation embryonic development, pregnancy success rate and embryo quality [60, 61].

The mechanism of CR keeping good egg quality is also shown in Fig. 3b. CR in adult mice upregulates mitochondrial SIRT3 in oocyte, and SIRT3 protect oocytes from the synthesis of mitochondrial reactive oxygen species (ROS). Therefore, high SIRT3 attenuates oxidative stress which declines oocyte quality with age [61, 62]. Also, CR in adult mice dramatically improves meiotic spindle assembly and maintenance, so it prevents oocyte aneuploid and chromosomal misalignment. In addition, CR enables to prevent the occurrence of aging-related mitochondrial dysfunction because it can appropriately arrange mitochondria in oocytes [61]. Although CR upregulates PGC-1α expression [60], Selesniemi et al. [61] discovered that loss of PGC-1α can reproduce the positive effect of CR on egg quality in aging female mice.

It is generally known that rodent models do not have true menses like humans. In humans, high size of PMF pool and slow rate of oocyte depletion are essential determinants of delayed menopause onset [69, 70]. As CR increases the number of PMFs and suppresses follicle development of rodents, it may have similar effect on human follicles. Therefore, CR seems to delay menopause onset and prolong reproductive lifespan of humans. However, the study of women who were exposed to Dutch famine discovered that CR decreases age at natural menopause, especially when occurring in early life [71]. The reason of this phenomenon is unknown. Another experiment showed an enigmatic discovery that the improvement of fecundity was observed in rabbits with CR alone [72]. Therefore, further studies are needed to explain these confusing findings.

### The roles of CR in ovulation

CR delays ovulation in mice [57], rhesus monkey [46], buffalo heifer [45] and women [73]. However, ovulation is increased in CR-then-AL-fed mice [7, 43]. There are two possible mechanisms of CR inhibiting ovulation. One is that in women CR reduces FSH secretion below the basal level. FSH deficiency cannot stimulate the growth of secondary follicles and thus the generation of dominant follicles where E_2_ is synthesized. Therefore, E_2_ concentration is too low to trigger an LH surge [74, 75]. In addition, low intra-ovarian IGF-1 caused by CR impedes E_2_ synthesis, thus inhibiting LH surge generation [35, 75, 76]. The other one found by Lujan et al. [46] is that CR inhibits gonadotropin surges in ovariectomized rhesus monkeys supplied with exogenous E_2_ and progesterone (P_4_), and the researchers summarized that CR inhibits gonadotropin surges in most CR-treated animals because CR impairs the hypothalamic response to the positive feedback of E_2_.

### The roles of CR in steroidogenesis

It has been discovered that CR reduces plasma E_2_. The discovery that chronic CR increase the expression of estrogen receptors but do not change the expression of androgen receptors in mice ovary also indicates the decreased level of serum E_2_ under CR [43]. Here we hypothesize that CR inhibits E_2_ synthesis. One possible mechanism is provided by the experiment in beef heifers [44]. Heifers with CR had lower plasma insulin, IGF-1 and LH, therefore STAR gene expression in theca cells is decreased. STAR transports cholesterol from the outer to the inner mitochondrial membrane, and then the intra-mitochondrial cholesterol can be converted into pregnenolone, resulting in E_2_ synthesis. As a result, CR-treated beef heifers reduced E_2_ production in dominant follicle. Another possible mechanism is related to intra-ovarian IGF-1/IGF-1R signaling. IGF-1 alone can increase the synthesis of E_2_, and it can synergistically activate FSH-induced aromatase that catalyzes the synthesis of E_2_ in rodents and humans [35, 75], so CR suppresses E_2_ synthesis. In addition, CR females have lower number of antral follicle where E_2_ is mainly synthesized.

It is less well-known about the effect of CR on P_4_ synthesis. CR reduces serum P_4_ in mice [56], buffalo heifer [45] and women [73]. However, it is found that there was no difference of follicular fluid P_4_ between beef heifers fed a diet of 1.2 times maintenance (M) and that fed a 0.4 M diet [44]. It is hypothesized that CR inhibits P_4_ production because CR females have less corpus luteum where most P_4_ is synthesized. In addition, low IGF-1 level may decrease P_4_ synthesis because IGF-1 alone or synergistically promotes P_4_ production [35].

## The roles of CR in the uterus

There are few experiments concentrating on the effect of CR on uterus. Basically, we can make sure that the reductions of serum E_2_ and P_4_ caused by CR impair endometrium development and function. The reason is that before ovulation E_2_ stimulates the rapid proliferation of endometrial stromal and epithelial cells. Also, E_2_ promotes the growth and vascularization of uterine glands. After ovulation, P_4_ produces a highly secretory endometrium and decidualizes the stromal cells to prepare an appropriate environment for implantation [74]. It is found that when women are exposed to CR during puberty, the mature GnRH neurons will become immature, increasing the risk of menstrual impairment [77]. The study in women proved that CR during reproductive age is related to irregular menses, and this deterioration becomes more serious if CR happens earlier. It is also discovered that CR during childhood prolongs the time from menarche to regular menses. However, CR during childhood seems not to negatively affect menstrual cycles in adulthood [78] (Table 1).

**Table 1:** The main roles of CR in uterus, pregnancy and reproductive-related diseases

Authors	Year	Species	Aspects	Influence of CR
Elias et al. [78]	2007	Humans	Uterus	CR during puberty relates to irregular menses, and CR during childhood prolongs the time from menarche to regular menses.
Lumey et al. [79]	1998	Humans	Pregnancy	CR in early pregnancy triggers compensatory growth of placenta.
Roseboom et al. [80]	2006	Humans	Pregnancy	Prenatal CR gives lasting negative consequences to offspring’s health, especially in early gestation.
Harper et al. [81]	2015	Mice	Pregnancy	CR during early gestation makes placental alternations reversible, resulting in metabolically normal offspring.
Harrath et al. [82]	2017	Rats	Pregnancy	Female offspring exposed to prenatal CR have an early puberty onset and a short reproductive lifespan.
Yarde et al. [83]	2013	Humans	Pregnancy	No relationship between prenatal CR and reproductive activities of offspring.
Fenichel et al. [84]	2007	Humans	Reproductive-related diseases	CR develops hypothalamic amenorrhea.
Marzouk et al. [85]	2015	Humans	Reproductive-related diseases	CR alleviates the deleterious conditions of PCOS patients with obesity.
Lope et al. [86]	2019	Humans	Reproductive-related diseases	CR reduces the incidence of breast cancer

*Notes*: *CR* caloric restriction, *PCOS* polycystic ovary syndrome

## The roles of CR in pregnancy

### Placenta is a critical hub in the effect of CR on offspring’s health

CR during pregnancy leads to maternal undernutrition (MUN). In mammals, MUN results in intrauterine growth restriction (IUGR) through reducing fetal nutrient availability, altering hormonal environment exposed to fetus and causing epigenetic changes in fetal genomes. These changes not only damage fetal health, but also increase the chronic disease susceptibility in postnatal life. Noticeably, placental alternation is a pivotal linkage of MUN to IUGR [87, 88].

Placenta is plastic to against exogenous insults. In women exposed to Dutch Famine during pregnancy, compensatory growth of placenta induced by MUN maintains consistent fetal nutrition to parturition, so the birthweight is normal [79] (Table 1). However, IUGR results if this adaptation alone cannot provide enough nutrients to ensure the normal fetal growth. In fact, impaired maternal-fetal circulation and nutrient transport system in placenta also mediate the influence of MUN on fetal development [87].

A series studies of humans who were exposed to Dutch Famine before birth discovered that MUN gives postnatal progeny the physical and cognitive impairments in life-long pattern. For example, MUN increases the prevalence of schizophrenia, coronary heart disease and type 2 diabetes. This deleterious effect is most obvious in early gestation, elucidating that early pregnancy is the most pivotal and vulnerable period [80, 89] (Table 1). The reason is that MUN in early pregnancy can permanently alter placenta, so it proceeds to affect the fetus till parturition, culminating in impairing postnatal health [81, 88]. In contrast, the observation in mice treated with 50% CR from days1.5–11.5 of pregnancy discovered that mice with CR could have reversible placental changes during early pregnancy, and their adult offspring was metabolically normal. It has been proposed that fetal development of humans expends more time than mice, so the timescale in humans is long enough to convert reversible compensation into irreversible overcompensation. This fact may support the phenomenon that humans have an irreversible but mice have a reversible placental alternation [81] (Table 1). It was summarized by Harper et al. [81] that the duration of changes in the placenta determines the duration of programming on fetus. The species differences in the effect of early-gestational MUN on adult phenotype are attributed to the extent of placenta recovery.

### Effects of prenatal CR in offspring’s reproduction

Intriguingly, MUN affects reproductive performances of animal offspring. Two experiments in rats [82, 90] found that female offspring exposed to prenatal CR had an aberrant ovarian follicle population, resulting in premature ovarian failure and reduced reproductive lifespan. Initially, these offspring had a significantly lower amount of PMFs and higher amount of primary follicles in prepubertal period. This observation indicates that in female descendants, PMF pool is affected by MUN during fetal life, and MUN advances the folliculogenesis, resulting in an early puberty onset. When these offspring reach adulthood, the number of PMFs and growing follicles (i.e. secondary follicles, antral follicles) were significantly reduced, suggesting that MUN causes a short reproductive lifespan. The reason is that the expression of genes, which are crucial for follicle maturation and ovulation, was reduced by both increased ovarian oxidative stress and impaired capacity for repairing oxidative damage [90]. Collectively, the female rat offspring born to mother with MUN have a more intensive and time-limited reproductive lifespan, and they can reproduce more successfully [82, 90] (Table 1). The experiment in sheep had a similar finding [91].

Regard to humans, Painter et al. [92] discovered a similar finding that women born to mother with MUN could reproduce more successfully if they were fed with improved nutrition in their postnatal period. However, Lumey et al. [93] raised an objection to Painter’s finding. He thought there was no difference in reproductive activities like delivery between women exposed to MUN during their fetal life and those not exposed to MUN during fetal life. And he attributed this inconsistent result to the fact that the Painter database was inappropriate and thus was not representative. In fact, Yarde et al. investigated mothers who were exposed to Dutch famine during gestation and discovered that MUN does not affect reproductive performances of offspring [83] (Table 1).

## The roles of CR in reproductive-related diseases

In women, CR enables to develop hypothalamic amenorrhea. The direct pathology is the impairment of HPO axis. The reduced GnRH secretion attenuates the gonadotropin secretion, therefore ovarian follicle development and E_2_ synthesis are inhibited. The insufficient E_2_ concentration cannot trigger the pre-ovulatory gonadotropin surges, culminating in anovulation and amenorrhea. Also, the reduced plasma leptin and the increased plasma ghrelin, which represent the low energy status, compromise the function of HPO axis and thus result in amenorrhea. Cognitive-behavioral therapy is considered as the possible treatment of amenorrhea, and E_2_ level is the index to assess the extent of HPO axis recovery [74, 84] (Table 1).

Approximately 5–10% reproductive-age women have polycystic ovary syndrome (PCOS), which is one of the commonest endocrine diseases. It is characterized by hyperandrogenism and chronic anovulation. Women with PCOS carry 2.7-fold increased risk of endometrial carcinoma [85, 94]. Noticeably, CR exerts a benefit effect on obese PCOS patients. In obese young adult women with PCOS, CR-induced weight loss ameliorates androgen overproduction, restores ovulatory cyclicity, improves menstrual function and attenuates insulin resistance. Therefore, dietary weight loss is considered to become the first-line treatment in obese PCOS patients [85, 95] (Table 1). Interestingly, giving CR in advance increases the survival rate of prepubertal obese/PCOS-prone rats when they encounter famine [96]. LH hypersecretion is observed in obese PCOS women, and CR usually attenuates pulsatile LH secretion in healthy women. However, daily LH secretion is still increased even these obese PCOS patients are treated with CR [97]. In addition, preserving E_2_-dependent negative feedback to LH can predict follicle maturation and ovulation in obese PCOS patients who are treated with CR [95].

In addition, another beneficial effect of CR displays on breast cancer. The study of women found that CR decreases the susceptibility of breast cancer. In contrast, excessive caloric intake increases the risk of developing BC. Researchers proposed that the combination of moderate CR and physical exercise is a prospective strategy to prevent breast cancer [86] (Table 1).

## Conclusion

In this review, we summarize that CR exerts both positive and negative effects on female reproduction system. CR impairs HPO axis and indeed reduces fertility in female mammals. Kisspeptin neuron is the crucial hub that links low energy state and HPO axis. In this review, there are three differences between rodents and humans. Firstly, CR in rodents simultaneously increases reproductive capacity and prolongs fertility lifespan. In contrast, CR advances the menopause onset of women. Secondly, placental alternation is reversible in mice while irreversible in women when CR takes place at pregnancy. Thirdly, prenatal CR shortens reproductive lifespan and increases fertility success in female rat offspring. However, it does not affect reproductive activities in human offspring. At last, we summarize that CR causes hypothalamic amenorrhea but ameliorates the deleterious condition of PCOS coupled with obesity. In addition, CR decreases the morbidity of breast cancer. The similarities and differences between animal and human results courage researchers to find the reasons behind them. Also, further studies focusing on human are needed.

## Data Availability

All data supporting the conclusion of this article are included in this published article.

## Abbreviations

AgRP: Agouti-related peptide
AL: Ad libitum
AMPK: AMP-activated protein kinase
α-MSH: α-melanocyte stimulating hormone
ARC: Arcuate nucleus
AVPV: anteroventral periventricular nucleus
BC: Breast cancer
CART: Cocaine- and amphetamine-regulated transcript
CR: Caloric restriction
CRH: Corticotropin releasing hormone
DYN: Dynorphin
E_2_: Estradiol
FSH: Follicle-stimulating hormone
GnRH: Gonadotropin-releasing hormone
HPO: Hypothalamus-pituitary-ovarian
IGF-1: Insulin-like growth factor
IGF-1Rs: IGF-1 receptors
IUGR: Intrauterine growth restriction
*kiss1*: Kisspeptin; Kiss1^ARC^: ARC kisspeptin neuron
Kiss1^AVPV^: AVPV kisspeptin neuron
*kiss1r*: Kiss1-receptor
mTORC1: mTOR complex 1
MC3R: Melanocortin receptor 3
MUN: Maternal undernutrition
NKB: Neurokinin B
NK3R: Neurokinin-3 receptor
NPY: Neuropeptide Y
NTS: Solitary tract nucleus
PCOS: Polycystic ovary syndrome
PeN: Periventricular nucleus
PMV: Premammillary nucleus
POA: Preoptic area
POMC: Pro-opiomelanocortin
PMFs: Primordial follicles
P_4_: Progesterone
LepRs: Leptin receptors
LH: Luteinizing hormone
ROS: Reactive oxygen species
rpS6: Ribosomal protein S6
RP3V: Rostral periventricular area of the third ventricle
S6K1: p70S6 kinase
4 V: The fourth ventricle
